# The impact of chromatin remodelling on cellulase expression in *Trichoderma reesei*

**DOI:** 10.1186/s12864-015-1807-7

**Published:** 2015-08-07

**Authors:** Thiago M. Mello-de-Sousa, Alice Rassinger, Marion E. Pucher, Lilian dos Santos Castro, Gabriela F. Persinoti, Rafael Silva-Rocha, Marcio J. Poças-Fonseca, Robert L. Mach, Roberto Nascimento Silva, Astrid R. Mach-Aigner

**Affiliations:** Department for Biotechnology and Microbiology, Institute of Chemical Engineering, TU Wien, Gumpendorfer Str. 1a, A-1060 Wien, Austria; Department of Biochemistry and Immunology, Ribeirão Preto Medical School, University of São Paulo 14049–900, Ribeirão Preto, SP Brazil; Laboratório Nacional de Ciência e Tecnologia do Bioetanol (CTBE), Centro Nacional de Pesquisa em Energia e Materiais (CNPEM), Campinas, São Paulo Brazil; Department of Genetics and Morphology, Institute of Biological Sciences, University of Brasilia, Brasília, DF Brazil

## Abstract

**Background:**

*Trichoderma reesei* is used for industry-scale production of plant cell wall-degrading enzymes, in particular cellulases, but also xylanases. The expression of the encoding genes was so far primarily investigated on the level of transcriptional regulation by regulatory proteins. Otherwise, the impact of chromatin remodelling on gene expression received hardly any attention. In this study we aimed to learn if the chromatin status changes in context to the applied conditions (repressing/inducing), and if the presence or absence of the essential transactivator, the Xylanase regulator 1 (Xyr1), influences the chromatin packaging.

**Results:**

Comparing the results of chromatin accessibility real-time PCR analyses and gene expression studies of the two prominent cellulase-encoding genes, *cbh1* and *cbh2,* we found that the chromatin opens during sophorose-mediated induction compared to D-glucose-conferred repression. In the strain bearing a *xyr1* deletion the sophorose mediated induction of gene expression is lost and the chromatin opening is strongly reduced. In all conditions the chromatin got denser when Xyr1 is absent. In the case of the xylanase-encoding genes, *xyn1* and *xyn2,* the result was similar concerning the condition-specific response of the chromatin compaction. However, the difference in chromatin status provoked by the absence of Xyr1 is less pronounced. A more detailed investigation of the DNA accessibility in the *cbh1* promoter showed that the deletion of *xyr1* changed the *in vivo* footprinting pattern. In particular, we detected increased hypersensitivity on Xyr1-sites and stronger protection of Cre1-sites. Looking for the players directly causing the observed chromatin remodelling, a whole transcriptome shotgun sequencing revealed that 15 genes encoding putative chromatin remodelers are differentially expressed in response to the applied condition and two amongst them are differentially expressed in the absence of Xyr1.

**Conclusions:**

The regulation of xylanase and cellulase expression in *T. reesei* is not only restricted to the action of transcription factors but is clearly related to changes in the chromatin packaging. Both the applied condition and the presence of Xyr1 influence chromatin status.

## Background

In nature, *Trichoderma reesei* is as a saprophytic fungus an excellent producer of enzymes involved in plant cell wall degradation (PCWD). In industry, these enzymes are used for a number of applications: xylanases are used for example in food industry as a baking agent and for clarification of juice and wine [1] or in the paper industry for de-inking [2]. Cellulases from *T. reesei* are important in textile industry for example for fibre polishing [3] or in the paper industry for recycling processes [2]. In the production of ethanol from cellulosic raw material *T. reesei* enzymes are applied to break down lignocellulose material to release D-glucose. The obtained D-glucose can be used subsequently in the sugar-to-ethanol fermentation (e.g. [4, 5] and citations therein). Due to the multiple applications of these enzymes many research studies have focused on this organism, its PCWD enzyme expression, and finally, the regulation of the encoding genes. Most of these studies were performed in the wild-type strain QM6a [6] or the mutant strain QM9414, which was selected for increased cellulase production [7]. Genome-wide analyses identified 34 cellulolytic and xylanolytic enzyme-encoding genes in *T. reesei* (reviewed in [8]), of which the most prominent cellulases are the cellobiohydrolases CBHI and CBHII (EC 3.2.1.91) [9] and the most studied xylanases are the endo-ß-1,4-xylanases XYNI and XYNII (EC 3.2.1.8) [10]. The mentioned research efforts led further to the identification of transcription factors involved in the regulation of the expression of genes coding for PCWD enzymes on the transcriptional level. The most important transactivator is the Xylanase regulator 1 (Xyr1), which is absolutely essential for expression of both, xylanase and cellulase-encoding genes [11]. However, it should be noted that only the cellulase expression strictly follows the induction/repression pattern of the *xyr1* gene [12]. The *xyr1* gene itself is usually expressed at a low level and can be induced by the disaccharide sophorose formed via transglycosylation [12, 13]. Otherwise, the xylanase expression depends on Xyr1, but the transcript levels of these genes do not strictly reflect *xyr1* transcript levels [11, 12]. The most important repressor is the Carbon catabolite repressor 1 (Cre1) [14], which mediates carbon catabolite repression (CCR) in presence of high amounts of easily usable carbon sources, such as D-glucose or D-xylose. Cre1 exerts its repressing function on both, the genes coding for the PCWD enzymes and the gene coding for their activator, *xyr1* (e.g. [13, 15]). The different response of *T. reesei*’s transcriptome and secretome to cellulose, sophorose, and D-glucose was just recently investigated in a comparative high-throughput genomic and proteomic study [16]. While a lot is known about the transcriptional regulation of *T. reesei*’s PCWD enzyme-encoding genes by regulatory proteins (reviewed in [17]), so far hardly anything was investigated concerning the impact of the chromatin status on their gene expression. Only for Cre1 it was already earlier suggested that it might influence chromatin remodelling [18]. More recently, it was reported that it is involved in nucleosome positioning [19], and that a truncated version of Cre1, which is present in CCR-released, cellulase hyper-producing strains, supports the opening of chromatin in Cre1-target genes [20]. However, taking into account that chromatin status generally is believed to be a crucial factor in gene expression, this topic did not receive much attention in *T. reesei* yet. Therefore, in this study, we aimed to learn if the opponent of Cre1, the transactivator Xyr1, is also involved in chromatin remodelling, and if this happens in a condition (inducing/repressing carbon source)-dependent way. We used chromatin accessibility real-time PCR (CHART-PCR) for determining the chromatin status of the genes encoding the mentioned, four major PCWD enzymes and compared this with their gene expression. The results prompted us to have a more detailed investigation of the *cbh1* promoter by *in vivo* footprinting analyses. Finally, we used whole transcriptome shotgun sequencing (WTSS) to identify genes putatively involved in chromatin remodelling that are differentially expressed with regards to the applied condition and/or the absence or presence of Xyr1.

## Results

### Decreased cellulase gene expression in the absence of Xyr1 goes along with denser chromatin

**Fig. 1:**
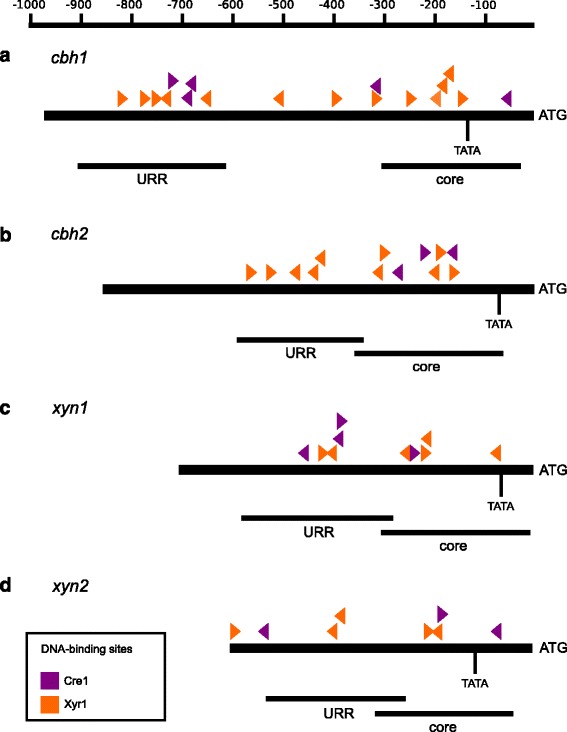
Overview on the upstream sequence of the investigated genes encoding PCWD enzymes. The regions investigated by CHART-PCR are indicated by black bars. The core promoter region covering the TATA-box (core) and an URR of the *cbh1* (**a**), *cbh2* (**b**), *xyn1* (**c**), and *xyn2* (**d**) genes each are depicted. DNA-binding sites of Xyr1 and Cre1 are indicated by orange and purple triangles, respectively. The orientation of the triangle represents the orientation of the binding motif. The scale at the top indicates distance from ATG in bp

**Fig. 2:**
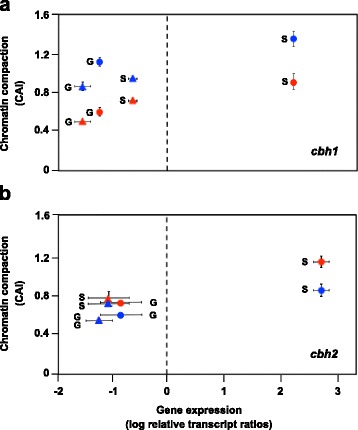
Transcript and CHART analysis of cellulase-encoding genes in the presence or absence of Xyr1. The *T. reesei* wild-type strain (dots) and the Δ*xyr1*-strain (triangles) were pre-grown on glycerol and thereafter incubated on D-glucose (G) or sophorose (S) for 3 h. The core promoter region (*red*) and an URR (*blue*) of *cbh1* (**a**) and *cbh2* (**b**) genes were investigated. The gene expression analysis was performed by cDNA synthesis followed by qPCR, and transcript levels are depicted on the x-axis. CHART-PCR was performed by DNaseI digestion followed by qPCR, and chromatin accessibility indices (CAIs) are depicted on the y-axis. In both cases *sar1* and *act* genes were used for data normalization and the wild-type strain incubated without carbon source for 3 h was the reference condition. The dashed line indicates transcript level of the reference condition, i.e. levels above are considered induced and levels below are considered repressed. All values are means from measurements in triplicates and three biological experiments (cultivations). The error bars indicate standard deviations. Diagrams are identically scaled

It is well known that Xyr1 is an essential activator of cellulase gene expression [11]. However, so far it has not been investigated if the deletion of Xyr1 additionally influences the chromatin status in the fungus. In order to study this, the wild-type strain and the *xyr1* deletion strain were pre-grown and transferred to sophorose (inducing condition), D-glucose (repressing condition) or no carbon source-containing medium (reference condition) and were incubated for 3 h. By applying CHART-PCR analysis we investigated the chromatin packaging of the core promoter region (bearing the TATA-box) and one upstream regulatory region (URR) bearing Xyr1-binding sites (5′-GGC(T/A)_3_-3′; [21]) and/or Cre1-binding sites (5′-SYGGRG-3′; [14]) of the *cbh1* and *cbh2* genes each. For overviews on the investigated regions see Fig. 1a, b. Supplementary, we investigated the transcript levels of these genes by reverse transcription, quantitative PCR (qPCR) to see if the expression is related to chromatin accessibility. The expression of *cbh1* and *cbh2* is repressed on D-glucose in both strains and induced by sophorose in the wild-type strain (Fig. 2a, b). The induction is lost in the *xyr1* deletion strain aside from a small increase in gene expression on sophorose compared to D-glucose. Altogether, we observed in both strains a condition-dependent change (i.e. sophorose-mediated opening) of chromatin that went along with a change (i.e. sophorose-mediated increase) in gene expression. However, comparing the strains under the same condition, the chromatin was always more closed in the *xyr1* deletion strain compared to the wild-type strain (Fig. 2a, b) indicating a contribution of Xyr1 to a general (i.e. condition-independent) opening of chromatin in upstream regions of the cellulase-encoding genes.

### Xylanase gene repression in the absence of Xyr1 is not strictly related to chromatin compaction

**Fig. 3:**
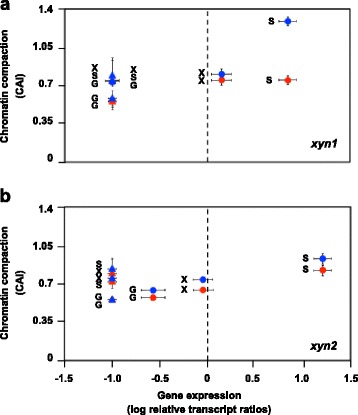
Transcript and CHART analysis of xylanase-encoding genes in the presence or absence of Xyr1. The *T. reesei* wild-type strain (dots) and the Δ*xyr1*-strain (triangles) were pre-grown on glycerol and thereafter incubated on D-glucose (G), D-xylose (X) or sophorose (S) for 3 h. The core promoter region (*red*) and an URR (*blue*) of *xyn1* (**a**) and *xyn2* (**b**) genes were investigated. The gene expression analysis was performed by cDNA synthesis followed by qPCR, and transcript levels are depicted on the x-axis. CHART-PCR was performed by DNaseI digestion followed by qPCR, and CAIs are depicted on the y-axis. In both cases *sar1* and *act* genes were used for data normalization and the wild-type strain incubated without carbon source for 3 h was the reference condition. The dashed line indicates transcript level of the reference condition, i.e. levels above are considered induced and levels below are considered repressed. All values are means from measurements in triplicates and three biological experiments (cultivations). The error bars indicate standard deviations. Diagrams are identically scaled

In an analogous analysis we investigated the chromatin status of the core promoter and an URR of the *xyn1* and *xyn2* genes each and compared this to the expression of the respective genes. For overviews on the regions investigated by CHART-PCR see Fig. 1c, d. In the wild-type strain the repression on D-glucose, the basal expression on D-xylose, and the induction on sophorose coincided with the increasing opening of chromatin (Fig. 3a, b). Otherwise, in the *xyr1* deletion strain the gene expression was at a similar low level (repressed) independent from the tested condition, while the chromatin packaging differed between the conditions. Interestingly, the chromatin accessibility on sophorose was even similar between the Δ*xyr1-*strain and the wild-type strain (except the URR of *xyn1*) but the sophorose-mediated induction was completely lost in the Δ*xyr1-*strain (Fig. 3a, b). Summarizing, we detected - similar to the case of the cellulase-encoding genes - an induction-specific opening of chromatin together with increasing gene expression in the wild-type strain. However, different from the cellulases, xylanase expression was repressed in the Δ*xyr1-*strain although the chromatin status differed condition-dependently.

### Contribution of Xyr1 to chromatin opening

**Fig. 4:**
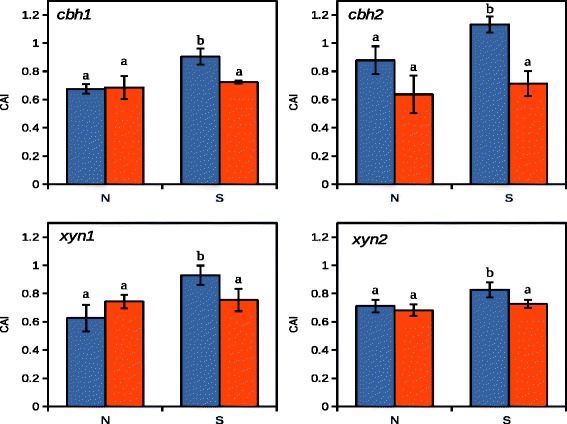
CHART analysis of cellulase- and xylanase-encoding genes in presence and. The *T. reesei* wild-type strain (*blue bars*) and the Δ*xyr1*-strain (*red bars*) were pregrown on glycerol and thereafter incubated without carbon source (N) or in presence of 2.0 mM sophorose (S) for 3 h. The core promoter regions of *cbh1*, *cbh2*, *xyn1*, and *xyn2* genes were investigated. CHART-PCR was performed by DNaseI digestion followed by qPCR using sar1 and act genes were for data normalization. Chromatin accession indices (CAI) are depicted on the y-axis. All values are means from measurements in triplicate and three biological experiments (cultivations). The error bars depict the standard deviation and different letters denote statistical difference among compared data employing t-test (*P* < 0.05)

To understand in detail the contribution of Xyr1 to changes in chromatin packing, the relation to induction of gene expression, and its putative impact on transcription initiation, we used CHART analysis again. We compared samples from the *T. reesei* wild-type and the *xyr1* deletion strain exposed to sophorose (inducing condition) and to non carbon source (non inducing condition). In the wild-type strain chromatin opens specifically on sophorose in case of all tested genes, namely *xyn1*, *xyn2*, *cbh1*, and *cbh2* (Fig. 4). This is lost for all genes in the Δ*xyr1-*strain (Fig. 4). However, the induction specific opening of chromatin is more pronounced in case of the cellulase-encoding genes. Altogether, the comparison of the chromatin accessibility under induced and non-induced conditions in the wild-type and the ∆*xyr1*-strain even suggested that the open status is a consequence of induction. Xyr1 is required for the chromatin loosening, but this action is not essential for the initiation of transcription because transcripts can also be detected at low levels in a *xyr1* deletion strain (compare Figs. 2 and 3).

### The absence of Xyr1 changes DNA accessibility in the *cbh1* promoter

**Fig. 5:**
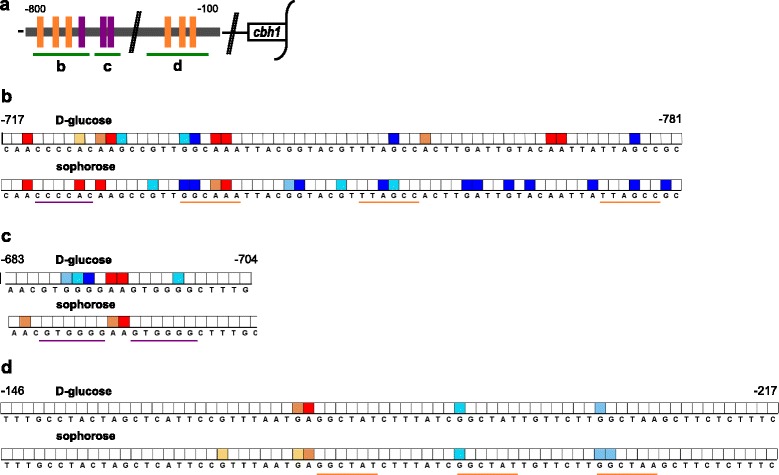
*In vivo* footprinting analyses of the *cbh1* promoter in the presence or absence of Xyr1. The *T. reesei* wild-type strain QM6a and the Δ*xyr1*-strain were pre-grown on glycerol and then incubated on D-glucose or sophorose for 3 h followed by DMS-induced *in vivo* methylation. **a** Schematic drawing of the *cbh1* promoter and the investigated regions (indicated by *green lines*). Two URRs (**b**, **c**) bearing functional Xyr1-binding sites (*orange*) or Cre1-sites (*purple*) and the core promoter region (**d**) bearing Xyr1-binding sites (*orange*) were investigated on the forward strand. Numbers indicate the position of the base upstream from ATG. Analysis of data and visualization was performed using ivFAST [34]. Only signals that are statistically different are considered; protected bases are highlighted in *red shades* and hypersensitive bases are highlighted in *blue shades*; the three colour intensities each correspond to stronger differences between compared conditions (Δ*xyr1-*strain compared to wild-type strain), i.e. increasing colour intensity means more than 1.1-, 1.3-, and 1.5-fold difference

Since we observed a pronounced induction-specific opening of chromatin that went along with increase of gene expression in presence of Xyr1 and a closing of chromatin together with gene repression in the absence of Xyr1 in the case of the cellulase-encoding genes, we aimed to have a more detailed investigation on the DNA accessibility of the promoter. Therefore, we performed *in vivo* footprinting analyses of the *cbh1* promoter. Two URRs bearing Xyr1-binding sites and/or Cre1-binding sites and the core promoter bearing Xyr1-binding sites close to the TATA-box were investigated (Fig. 5a). The wild-type strain and the Δ*xyr1-*strain were pre-grown on glycerol and then incubated on D-glucose or sophorose for 3 h followed by dimethyl sulphate (DMS)-induced *in vivo* methylation. From Fig. 5b–d the footprinting pattern of the *xyr1* deletion strain compared to the wild-type strain for the three investigated regions can be inferred. The first investigated URR bears next to a single Cre1-site and a single Xyr1-site, also two Xyr1-sites arranged as inverted repeat with a spacing of 11 bp, which was reported to be the functional binding motif *in vivo* [22]. Under both, repressing and inducing conditions we could detect strong differences in the footprinting pattern of the two strains (Fig. 5b). In particular on sophorose, we observed an increased hypersensitivity towards DNA methylation on the Xyr1-sites in the Δ*xyr1-*strain compared to the wild-type strain, whereas the Cre1-site was stronger protected (Fig. 5b). The second investigated URR bears a functional Cre1 double site [23]. Here, we detected strong hypermethylation signals in the Δ*xyr1-*strain compared to the wild-type strain on D-glucose, but none on sophorose (Fig. 5c). The third investigated URR bears three Xyr1-binding sites arranged *in tandem*. In this case, we detected just a few differences between the two strains, however, most of them on or close to the Xyr1-sites (Fig. 5d).

### Identification of differentially expressed genes potentially involved in chromatin remodelling

To learn more about the mechanisms responsible for the chromatin remodelling in context to both, the applied condition and the presence or absence of Xyr1, we used WTSS. Therefore, a *xyr1* deletion strain and its parental strain QM9414 were again exposed to repressing conditions (growth on D-glucose) and inducing conditions (incubation on sophorose). Please note that the full data set can be obtained from GEO database (GSE66982). Based on the results obtained by the WTSS, we analyzed the gene expression profiles of 136 candidate genes involved in chromatin structure and dynamics according to the eukaryotic orthologous groups (KOG) in the *T. reesei* genome database (http://genome.jgi-psf.org/Trire2/Trire2.home.html). An overview on these 136 genes is provided here. For the differential expression analysis, a two-fold change cut-off, *i.e.* log_2_ fold change ≥ 1 or ≤ −1 and an adjusted *p*-value ≤ 0.05, was used as threshold. Concerning the first part of our question, *i.e.* the observed differences in chromatin status dependent on the applied condition, we identified 15 genes differentially expressed on sophorose as compared to D-glucose in the wild-type strain (listed in Table 1). Concerning, the second part of our question, i.e. the influence of Xyr1 on the expression profiles of these genes we examined which ones were differentially expressed in Δ*xyr1*-strain compared to the wild-type strain under sophorose induction. Out of the 15 genes responding to the applied condition, two genes are additionally differentially expressed in the Δ*xyr1*-strain (transcript ID 53947 and 73708). Notably, the gene with transcript ID 73708, encoding a putative heterochromatin-associated protein, was down-regulated on sophorose compared to D-glucose and up-regulated in the absence of Xyr1.

**Table 1:** Differentially expressed genes that are potentially involved in chromatin remodelling

Transcript ID	Annotation	SO/G	*p*-value	Δ*xyr1*/WT	*p*-value
2648	Predicted component of NuA3 histone acetyltransferase complex	−1.154	0.000	0.279	0.653
34402	Histone H1	1.300	0.000	−0.457	0.075
36727	SWI-SNF chromatin-remodeling complex protein	1.493	0.000	−0.843	0.000
53947	SWI-SNF chromatin-remodeling complex protein	1.196	0.000	1.332	0.000
56077	SWI-SNF chromatin-remodeling complex protein	3.050	0.000	−0.359	0.158
65533	Histone deacetylase complex, catalytic component HDA1	1.237	0.000	−0.347	0.170
73708	Heterochromatin-associated protein HP1 and related CHROMO domain proteins	−3.012	0.002	1.253	0.000
76872	SWI-SNF chromatin-remodeling complex protein	1.070	0.000	−0.708	0.004
81517	Sirtuin 5 and related class III sirtuins (SIR2 family)	1.600	0.034	−0.160	0.615
108909	Nucleosome-binding factor SPN, POB3 subunit	1.050	0.000	0.041	0.998
110409	Possible homologue of *S. cerevisiae* SAS10	−1.298	0.000	−0.001	1.000
110418	SWI-SNF chromatin-remodeling complex protein	1.180	0.000	−0.566	0.049
110507	Histone acetyltransferase (MYST family)	1.064	0.000	−0.562	0.024
122943	SWI-SNF chromatin-remodeling complex protein	1.876	0.000	−0.127	0.621
123327	SWI-SNF chromatin-remodeling complex protein	1.852	0.002	0.508	0.036

Differential gene expression according to WTSS analysis comparing either sophorose induction (SO) to glucose repression (G) in the wild-type strain or the *xyr1* deletion strain (Δ*xyr1*) to the wild-type strain (WT) under sophorose induction

## Discussion

The aim of this study was to learn more about the contribution of the chromatin compaction to the regulation of gene expression of PCWD enzymes in *T. reesei*. Altogether, we found for all investigated genes that their induced expression is accompanied by an opening of chromatin and that Xyr1 is required for the open chromatin status.

However, we observed differences between cellulase- and xylanase-encoding genes concerning the involvement of Xyr1 in chromatin remodelling: the chromatin of the upstream regions of the cellulase-encoding genes was more compact under all tested conditions when Xyr1 was missing. This finding is supported by the *in vivo* footprinting results of the *cbh1* URR, which revealed an increased sensitivity towards methylation on the Xyr1-binding sites in the absence of Xyr1, in particular on sophorose (compare Fig. 4b). On the other hand, the accessibility of the functional Cre1-sites was changed only on D-glucose. They were found to be stronger methylated in the *xyr1* deletion strain than in the parent strain (compare Fig. 4c). We assume that hypersensitivity to DNA methylation can be caused by both, non-occupancy leading to better access for the methylation agent, but also by DNA occupancy and a following increased disposition to be methylated. Considering this, we would suggest that on D-glucose repression the Cre1 DNA-binding affinity to the *cbh1* promoter is higher in the absence of Xyr1. This could explain the less accessible chromatin in the ∆*xyr1*-strain on D-glucose (compare Fig. 2a). In summary, the presence of Xyr1 supported chromatin opening under all investigated conditions in the case of the cellulase-encoding genes. Perhaps this finding is one explanation for the previously reported, condition-dependent transcript level pattern of the *cbh1* and *cbh2* genes that exactly follow the one of the *xyr1* gene [12]. For example, if under non-inducing conditions less Xyr1 is present, the positive influence of Xyr1 on chromatin opening might be reduced and this would cause a decrease in cellulase-encoding gene expression. However, the earlier observation that transcript levels of *cbh1* and *cbh2* correlate with those of *xyr1* [12], and the result from this study indicating the involvement of Xyr1 in chromatin opening suggest a regulation of the cellulase-encoding genes being dominated by Xyr1.

It is currently thought that transcription factors must induce the reorganization of the local chromatin (reviewed by [24]). One proposed mechanism is the recruitment of nucleosome remodelers by the initiating factor leading to local chromatin conformations [25]. Our current model on the function of the Xyr1 is the following: as shown in previous studies *xyr1* transcription is induced on sophorose [12]. This allows the assumption that under this condition Xyr1-sites are occupied, which is supported by *in vivo* footprinting results obtained during this study. We would suggest that Xyr1 recruits chromatin remodelers leading to the observed, more open chromatin status. This provides easier access for the transcription machinery leading to increased induction of the target gene (i.e. the cellulase-encoding gene) under this condition.

In the case of the xylanase-encoding genes we also detected a condition-dependent induction of gene expression, which was accompanied by chromatin opening in the wild-type strain. However, the involvement of Xyr1 is different in this case as compared to the cellulase-encoding genes. In the absence of Xyr1 gene expression decreased under all conditions, but the chromatin in the upstream regions of the xylanase-encoding genes did not always became more compact. For example, in the case of *xyn2,* the URR had a similar chromatin accessibility under non-repressing conditions (sophorose, D-xylose) in the Δ*xyr1-*strain as in the wild-type strain but the gene expression was strongly repressed in the absence of Xyr1. The fact that gene expression can be repressed simultaneously with enhanced chromatin accessibility might be explained by a generally better access for all kinds of regulatory proteins including repressor proteins. Another possible explanation would be that the absence of Xyr1 simply overrules the level of regulation by chromatin opening. Anyway, during this study it became obvious that the activating function of Xyr1 on xylanase-encoding gene expression is not mainly exerted on the chromatin level. There are earlier reports on generally different, condition-dependent transcript level patterns of the *xyn1* and *xyn2* genes as compared to the *xyr1* gene [12]. One example is the low basal *xyn2* gene expression on D-glucose (e.g. [26, 27]) that is not detectable for the *xyr1* gene [13]. All these findings together strongly indicate that additional regulatory factors (for example the suggested xylanase repressor Xpp1 [28]) and mechanisms, which are responsible for chromatin opening under inducing conditions, need to be involved.

A whole transcriptome analysis was used to identify genes classified as chromatin remodelers in *T. reesei*, which are differentially expressed dependent on the applied condition (inducing/repressing). Notably, 15 genes are differentially expressed in the wild-type strain (compare Table 1), whereas only ten genes responded in a condition-dependent manner in the Δ*xyr1*-strain (data not shown). This again supports the assumption that Xyr1 is generally involved in chromatin remodelling mechanisms. The identification of two putative chromatin remodelers, which are under the control of Xyr1 (directly or via expression of other regulatory proteins), point to an indirect role of Xyr1 in chromatin remodelling. Moreover, it can be speculated that Xyr1 additionally recruits chromatin-remodelling proteins in a differential manner towards the promoters of the cellulase- and xylanase-encoding genes. This would be a further explanation for the observed differences concerning the influence of Xyr1 on their chromatin status. However, at this stage it remains to be investigated if the open chromatin is indeed the result of chromatin remodelling (as the loss or movement of nucleosomes) or if the loss of the identified putative chromatin remodelers overrules the action of Xyr1.

## Conclusions

Investigations on the level of chromatin packaging revealed that the transcription factor Xyr1 does exert its activating function—in addition to other possible mechanisms - by an induction-specific opening of chromatin. The impact of Xyr1 in chromatin opening was more pronounced in the case of cellulase-encoding genes than in the case of the xylanase-encoding genes. The application of WTSS identified one chromatin remodeler that is down-regulated under inducing conditions and up-regulated if Xyr1 is missing. According to the results of the present study, this is a target in engineering strains with enhanced cellulase expression.

## Methods

### Fungal strains

The following *T. reesei* strains were used throughout this study: the wild-type strain QM6a (ATCC 13631), and a corresponding *xyr1* deletion strain (this study), QM9414 (ATCC 26921), and a QM9414 strain bearing a *xyr1* deletion [11]. All strains were maintained on malt extract agar.

### Growth conditions

For carbon source replacement experiments mycelia were pre-cultured in 1-L-Erlenmeyer flasks on a rotary shaker (180 rpm) at 30 °C for 24 h in 250 mL of Mandels-Andreotti (MA) medium [29] supplemented with 1 % (w/v) glycerol as sole carbon source. A total of 10^9^ conidia per litre (final concentration) were used as inoculum. Pre-grown mycelia were washed and equal amounts were resuspended in 20 ml MA media containing 1 % (w/v) D-glucose or 2 mM sophorose as sole carbon source or no carbon source and were incubated for 3 h.

For direct cultivation experiments conidia were incubated in 200 mL MA medium containing 2 % (w/v) glucose as the sole carbon source for 24 and 48 h. Samples were derived from three biological replicates.

### Deletion of *xyr1* from the genome of the *T. reesei* wild-type strain

The deletion of the *xyr1* gene was essentially performed as described in [11]. The plasmid pD2xlr1 was modified by shortening the promoter of the *A. nidulans amdS* gene, which was used as a marker [30]. The obtained plasmid pD5 was applied in a fungal protoplast transformation using QM6aΔ*tmus53* [31] as a recipient strain and was performed by following the protocol described in [32].

### CHART-PCR

DNase I digestion of chromatin and DNA extraction were carried out as described before [20]. qPCR analysis of the DNase I-treated samples was performed to measure the relative abundance of target regions. PCRs were performed in triplicates in a Rotor-Gene Q system (Qiagen, Hilden, Germany) using the amplification mixture (final volume 20 μL) and cycling conditions described before [20]. Primer sequences are provided in Table 2. The amount of intact input DNA of each sample was calculated by comparing the threshold values of the PCR amplification plots with a standard curve generated for each primer set using serial dilutions of genomic, undigested DNA. The chromatin accessibility index (CAI) was defined as: CAI = 1/(Ds/((Dc1 + Dc2)/2)), where Ds is the amount of intact DNA detected for each target region and Dc1 and Dc2 are the amounts of intact DNA detect for the promoter regions of *sar1* and *act* respectively, used as reference genes for normalization.

**Table 2:** Oligonucleotides used in this study

Name	Sequence (5′ - 3′)	Usage
RG53	GAATTCAGATC	ivFP, oligo-short
RG54	GCGGTGACCCGGGAGATCTGAATTC	ivFP, oligo-long
RG83	[6-FAM]CCTTTGGGTGTACATGTTTGTGCTCCGG	ivFP, cbh1oligo3fw
RG84	[6-FAM]GGAGAGTGCAGGCCGACTGAGC	ivFP, cbh1oligo3rev
RG89	[6-FAM]GTAGAGGCATGTTGTGAATCTGTGTCGGG	ivFP, cbh1oligo3fw
RG90	[6-FAM]GGTTGTATGCAAAACGCTCCGAGTCAGAC	ivFP, cbh1oligo3rev
actfw	TGAGAGCGGTGGTATCCACG	qPCR
actrev	GGTACCACCAGACATGACAATGTTG	
sar1fw	TGGATCGTCAACTGGTTCTACGA	
sar1rev	GCATGTGTAGCAACGTGGTCTTT	
cbh1f	GATGATGACTACGCCAACATGCTG	
cbh1r	ACGGCACCGGGTGTGG	
cbh2f	CTATGCCGGACAGTTTGTGGTG	
cbh2r	GTCAGGCTCAATAACCAGGAGG	
xyn1f	CAGCTATTCGCCTTCCAACAC	
xyn1r	CAAAGTTGATGGGAGCAGAAG	
taqxyn2f	GGTCCAACTCGGGCAACTTT	
taqxyn2r	CCGAGAAGTTGATGACCTTGTTC	
epiactinTr_f	CTTCCCTCCTTTCCTCCCCCTCCAC	act CHART, region −226 to +24
epiactinTr_r	GCGACAGGTGCACGTACCCTCCATT	
episar1Tr_f	GTCAGGAAATGCCGCACAAGCAAGA	sar1 CHART, region −490 to −224
episar1Tr_r	TGTGTTTTACCGCCTTGGCCTTTGG	
epicbh1_1Tr_f	AAGGGAAACCACCGATAGCAGTGTC	cbh1 CHART, region −902 to −610
epicbh1_1Tr_r	TTTCACTTCACCGGAACAAACAAGC	
epicbh1_2Tr_f	GGATCGAACACACTGCTGCCTTTAC	cbh1 CHART, region −301 to −27
epicbh1_2Tr_r	GGTTTCTGTGCCTCAAAAGATGGTG	
epicbh2_1Tr_f	CGGATCTAGGGCAGACTGGGCATTG	cbh2 CHART, region −587 to −338
epicbh2_1Tr_r	GTGTAGTGTTGCGCTGCACCCTGAG	
epicbh2_2Tr_f	TGCAGCGCAACACTACACGCAACAT	cbh2 CHART, region −355 to −62
epicbh2_2Tr_r	TGCGCCTCATACAGGGTCACAGTCC	
epixyn1_1Tr_f	GCACTCCAAGGCCTTCTCCTGTACT	xyn1 CHART, region −577 to −278
epixyn1_1Tr_r	TAGATTGAACGCCACCCGCAATATC	
epixyn1_3Tr_f	GTCGATATTGCGGGTGGCGTTCAAT	xyn1 CHART, region −306 to −10
epixyn1_3Tr_r	TTTGTGCGTGTTTTCCTTGAAGTCG	
epixyn2_1Tr_f	GTGCCGATGAGACGCTGCTGAGAAA	xyn2 CHART, region −527 to −252
epixyn2_1Tr_r	GATATTGCGCCTTGCAACACCATCG	
epixyn2_2Tr_f	CTCGAGACGGCTGAGACAGCAGCAT	xyn2 CHART, region −311 to −38
epixyn2_2Tr_r	TGTCTTTTGGGCTTGGAGGGGTTGT	

### Analysis of transcript levels

Fungal mycelia were homogenized in 1 mL of peqGOLDTriFast DNA/RNA/protein purification system reagent (PEQLAB Biotechnologie, Erlangen, Germany) using a FastPrep(R)-24 cell disrupter (MP Biomedicals, Santa Ana, CA, USA). RNA was isolated according to the manufacturer’s instructions, and the concentration was measured using the NanoDrop 1000 (Thermo Scientific, Waltham, US). Synthesis of cDNA from mRNA was carried out using the RevertAidTM H Minus First Strand cDNA Synthesis Kit (Thermo Fisher Scientific) according to the manufacturer’s instructions. Quantitative PCRs were performed in triplicates in a Rotor-Gene Q system (Qiagen). The amplification mixture (final volume 15 μL) contained 7.5 μL 2 × iQ SYBR Green Mix (Bio-Rad, Hercules, USA), 100 nM forward and reverse primer and 2.5 μL cDNA (diluted 1:20). Primer sequences are provided in Table 2. Cycling conditions and control reactions were performed as described previously [33]. Data normalization using *sar1* and *act* as reference genes and calculations were performed as published previously [33].

### *In vivo* footprinting

*In vivo* methylation using DMS followed by ligation mediated PCR was performed as described previously [34]. FAM-labelled fragments were generated by a PCR reaction using RG89 and RG90 or RG83 and RG84 for an URR or a TATA-box containing core region within the *cbh1* promoter, respectively. Primer sequences are provided in Table 2. FAM-labelled fragments were analyzed by capillary gel electrophoresis (Microsynth, Balgach, Switzerland) and results were analyzed using the program ivFAST [34].

### Whole transcriptome shotgun sequencing

The mRNA was extracted from fungal mycelia using TRIzol^®^ RNA Kit (Life Technologies, part of Thermo Fisher Scientific, Waltham, MA, USA) according to the manufacturer’s instructions. RNA concentration was determined by spectrophotometry at 260/280 nm and RNA integrity was tested by the 2100 Bioanalyzer (Agilent Technologies, Santa Clara, CA, USA) and gel electrophoresis (1 % agarose). The RNA of the biological replicates was pooled, lyophilized, and stored using the RNAstable^®^ Tube Kit (Biomatrica, San Diego, CA, USA). Barcoded libraries were prepared using the TruSeq RNA Sample Prep kit (Illumina, San Diego, CA, USA) and sequenced by LGC Genomics GmbH (Berlin, Germany) using the Illumina HiSeq 2000 platform.

### WTSS data analysis

Sequences from approximately 144 million 100 bp paired-end reads were quality-filtered and mapped to the *Trichoderma reesei* 2.0 reference genome, (http://genome.jgi-psf.org/Trire2/Trire2.home.html) using the Bowtie aligner version 0.12.8 [35] allowing two mismatches and only unique alignments. The SAMtools version 0.1.18 [36] was used to process the alignments files, which were visualized using the Integrative Genomics Viewer [37]. Bioconductor DESeq package version 1.10.1 [38] was utilized for normalization, using the median log deviation, and for the differential expression analysis, applying a two-fold change cut-off, i.e. log_2_-fold change ≥ 1 or ≤ −1 and an adjusted *p*-value ≤ 0.05 were used as thresholds. The log_2_-fold change was calculated according to the equation:

$$ { \log}_2-\mathrm{fold}\ \mathrm{change} = { \log}_2\ \frac{baseMeanB}{baseMeanA},\;\mathrm{where}: $$

baseMeanB is the mean normalized counts from condition B and baseMeanA is the mean normalized counts from condition A.

## Abbreviations

CAI: Chromatin accessibility index
CCR: Carbon catabolite repression
CHART-PCR: Chromatin accessibility real-time PCR
Cre1: Carbon catabolite repressor 1
DMS: Dimethyl sulphate
ivFP: *in vivo* footprinting
KOG: Eukaryotic orthologous groups
MA: Mandels-Andreotti
PCWD: Plant cell wall-degrading/plant cell wall degradation
qPCR: Quantitative PCR
URR: Upstream regulatory region
WTSS: Whole transcriptome shotgun sequencing
Xyr1: Xylanase regulator 1
